# Early infant HIV-1 diagnosis programs in resource-limited settings: opportunities for improved outcomes and more cost-effective interventions

**DOI:** 10.1186/1741-7015-9-59

**Published:** 2011-05-20

**Authors:** Andrea L Ciaranello, Ji-Eun Park, Lynn Ramirez-Avila, Kenneth A Freedberg, Rochelle P Walensky, Valeriane Leroy

**Affiliations:** 1 Division of Infectious Disease, Massachusetts General Hospital, Boston, MA, USA; 2 Division of General Medicine, Massachusetts General Hospital, Boston, MA, USA; 3 Division of Infectious Diseases, Children's Hospital Boston, Boston, MA, USA; 4 Center for AIDS Research, Harvard Medical School, Boston, MA, USA; 5 Division of Infectious Disease, Brigham and Women's Hospital, Boston, MA, USA; 6 Inserm, Unité 897, Institut de Santé Publique, Epidémiologie et Développement (ISPED), Université Bordeaux Segalen, Bordeaux, France

## Abstract

Early infant diagnosis (EID) of HIV-1 infection confers substantial benefits to HIV-infected and HIV-uninfected infants, to their families, and to programs providing prevention of mother-to-child transmission (PMTCT) services, but has been challenging to implement in resource-limited settings. In order to correctly inform parents/caregivers of infant infection status and link HIV-infected infants to care and treatment, a 'cascade' of events must successfully occur. A frequently cited barrier to expansion of EID programs is the cost of the required laboratory assays. However, substantial implementation barriers, as well as personnel and infrastructure requirements, exist at each step in the cascade. In this update, we review challenges to uptake at each step in the EID cascade, highlighting that even with the highest reported levels of uptake, nearly half of HIV-infected infants may not complete the cascade successfully. We next synthesize the available literature about the costs and cost effectiveness of EID programs; identify areas for future research; and place these findings within the context of the benefits and challenges to EID implementation in resource-limited settings.

## Introduction

Mother-to-child transmission (MTCT) of HIV-1 results in approximately 370,000 infant infections worldwide each year [1]. HIV-1 early infant diagnosis (EID) programs seek to inform the caregivers of HIV-exposed infants of infant HIV infection status, and to link HIV-infected infants to care and treatment. EID of HIV confers substantial benefits to infants and families, both for HIV-infected and uninfected infants, as well as to programs providing prevention of mother-to-child transmission (PMTCT) services. The World Health Organization (WHO) estimates that only 6% to 15% of HIV-exposed infants under 1 year of age accessed EID programs in 2008-2009 [2], highlighting that the implementation of effective EID programs has been challenging in resource-limited settings and demands careful attention.

**Figure 1:**
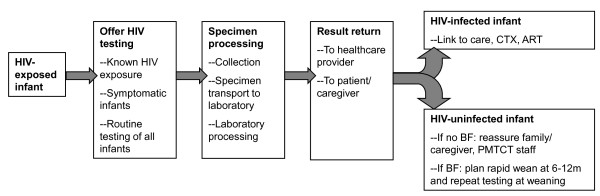
**Early infant diagnosis (EID) cascade: where could interventions be most effective and cost effective? **The cascade of care required for optimally effective EID programs, with two primary goals: (1) correctly informing caregivers of infant infection status and (2) linking all HIV-infected infants to care and antiretroviral therapy (ART). BF = breastfeeding; CTX = cotrimoxazole; PMTCT = prevention of mother-to-child HIV transmission.

A frequently cited barrier to expansion of EID programs is the availability and cost of the required laboratory assays, which are usually PCR based and therefore more expensive than the antibody-based testing used for older children and adults [3,4]. However, cost is not the only barrier to implementing EID programs. Opportunities to optimize infant outcomes may be lost at each step in a 'cascade' of EID and pediatric HIV care (Figure 1), conceptually similar to the recently described 'cascade' of care required for effective PMTCT services [5]. The EID cascade includes the offer and acceptance of EID testing among HIV-exposed infants, including those for whom HIV exposure was unknown; accurate specimen collection, transport, and laboratory processing; relay of results to both healthcare providers and infants' families/caregivers; and linkage to care, cotrimoxazole prophylaxis, and antiretroviral therapy (ART) for infants identified as HIV infected. In this review, we outline the ways in which interventions targeting each step in this EID cascade may impact clinical outcomes, costs, and cost effectiveness of EID programs.

## Currently available assays for early infant diagnosis

**Figure 2:**
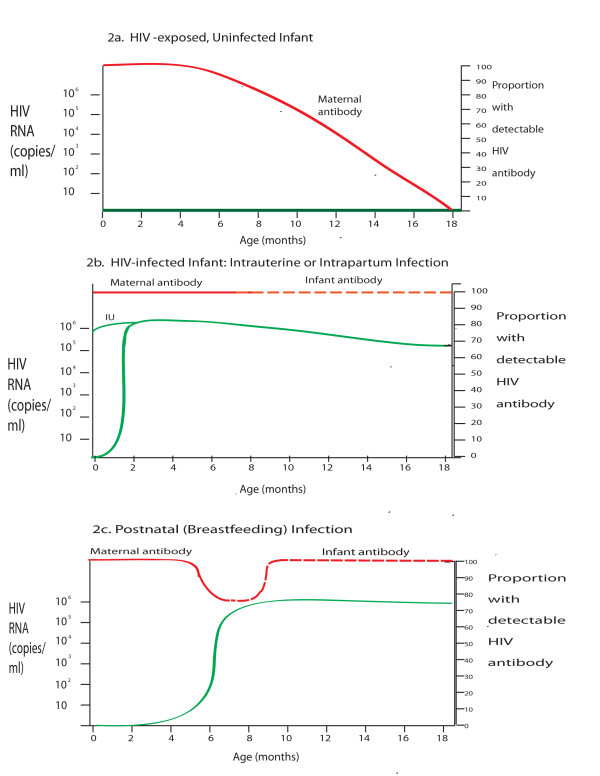
**HIV RNA levels and anti-HIV antibody responses among HIV-exposed infants with and without HIV infection**. Schematic depiction of the timing of positive HIV-1 antibody testing and RNA levels among HIV-exposed infants. The horizontal axis shows infant age in months. The left vertical axis shows mean HIV-1 RNA level on a logarithmic scale, and corresponds to the green lines on each graph. The right vertical axis shows the proportion of infants for whom an HIV antibody test would likely return positive, and corresponds to the red lines on each graph. The proportion of infants with a positive antibody test in all panels is approximate, based on a wide range of reported ages at which transmitted maternal HIV antibody fades from detection in the sera of uninfected infants [7,105]. Similarly, the mean RNA level is also approximate, based on several studies of infected infants with and without receipt of antiretroviral drugs for prevention of mother-to-child HIV transmission (PMTCT) [106-109]. **(a) **Results for an HIV-exposed infant who is born without HIV infection and remains uninfected throughout breastfeeding. In this case, HIV-RNA level remains zero, and maternal HIV antibody fades with time. **(b) **Results for infants infected before birth, either during the intrauterine period (IU; resulting in a high RNA level immediately after birth) or during the intrapartum period (IP; resulting in a 1-2 week delay before viremia is detectable). Maternal HIV antibody is present at birth; although maternal antibody fades with time, endogenous infant antibody production begins in response to infant infection. **(c) **Results for an HIV-exposed infant who is uninfected at birth, but becomes infected at approximately 6 months of age through breastfeeding. HIV RNA is undetectable while the infant is uninfected, but rises rapidly within the first few weeks after infection. Maternal antibody is present at birth and begins to fade with time, but infant antibody production begins after infant infection occurs.

In adults and older children, chronic HIV-1 infection can be diagnosed accurately by detection of serum antibodies against HIV-1 [6]. However, in infants born to HIV-infected women, maternal anti-HIV antibodies cross the placenta and persist in infant blood for up to 18 months (Figure 2). When detected in young infants, these antibodies usually represent exposure to maternal HIV rather than true infant HIV infection [7,8]. Antibody-based tests can therefore be accurately used only to exclude infant infection after 12 months of age, or to confirm infant infection after 15-18 months of age [6,9].

Accurate EID before 12-18 months of age therefore requires detection of viral components in infant blood ('virological testing'), including cell-free RNA, DNA integrated into host cells, or the viral capsid p24 antigen. Both RNA and DNA can be detected by PCR-based assays. DNA PCR assays, which provide a qualitative result, have traditionally been preferred for EID over quantitative RNA PCR assays, which are more often used for viral load monitoring after established diagnosis. This preference for DNA PCR assays developed for two primary reasons: (1) DNA assays require whole blood, which is easier to obtain than the plasma required for RNA assays, and (2) early-generation DNA assays demonstrated greater sensitivity than RNA assays if mothers or infants received antiretroviral drugs for PMTCT [10-14]. Multiple RNA and DNA PCR assays are now used for EID in resource-limited settings; the sensitivity, specificity, and limitations of these assays have been well summarized [11,12,15-19]. In addition to PCR assays, ultrasensitive (immune complex-dissociated) p24 antigen assays may soon represent an accurate, low-cost method for EID [8,11,15,20-27]. Furthermore, the use of dried blood spots (DBS; whole blood obtained via heel stick or finger prick and dried on filter paper) has obviated phlebotomy requirements for all three assay types. Because DBS specimens are heat stable, non-infectious, and can be shipped via mail or courier, DBS sampling has also reduced the cost of specimen transport to laboratories with PCR or p24 processing capacity [8,20,21,28-32].

## Assays for early HIV diagnosis among breastfed infants

Breastfed infants are at ongoing risk of postnatal HIV acquisition [6]. A positive virological test result in a breastfed infant indicates infant HIV infection; however, virological tests may be falsely negative immediately after postnatal infection has occurred (Figure 2). HIV care and treatment guidelines therefore recommend that HIV infection in an HIV-exposed infant cannot be definitively excluded until at least 6 weeks after cessation of breastfeeding [10]. Additionally, breastfeeding in many settings may continue through the first 2-3 years of life, suggesting an important role for repeat testing, both during the breastfeeding period, to detect incident infant infections, and at least 6 weeks after weaning, to confirm HIV-negative diagnoses.

## Benefits of early infant diagnosis

The Children with HIV Early ART (CHER) trial in South Africa recently demonstrated a 76% reduction in morbidity, a 75% reduction in mortality, and notable short-term cost reductions when HIV-infected infants initiated ART before 3 months of age and before clinical signs and symptoms of HIV developed [33,34]. Based on this trial and a growing body of observational data [35-37], WHO pediatric treatment guidelines now recommend ART initiation for all HIV-infected infants under 24 months of age [6,32]. In order to facilitate ART initiation as soon as possible after infection, WHO recommends HIV diagnostic testing for all HIV-exposed infants (infants born to HIV-infected mothers) at 4-6 weeks of age [32].

EID confers many potential benefits in addition to early ART initiation. As PMTCT programs are expanded, the majority of HIV-exposed infants will be uninfected and negative virological tests in infancy will provide reassurance to families [11]. In addition, when HIV-exposed infants are raised by extended families due to maternal disability or death, caregivers may bond more readily to infants known to be HIV negative [38]. Documented infant HIV status also permits assessment of PMTCT program effectiveness, and the recognition that a large proportion of HIV-exposed infants are uninfected has improved morale among PMTCT program staff [11].

Accurate EID also informs infant feeding decisions in settings where breastfeeding is recommended for improved infant health. For example, if infants are found to be HIV infected, WHO recommends breastfeeding for 2 years or longer, to avoid the increased incidence of diarrhea, pneumonia, and death observed with replacement feeding [39,40]. A diagnosis of HIV infection also permits discontinuation of postnatal antiretroviral prophylaxis, reducing the risk of drug-resistant virus associated with these medications [41,42]. If infants are found to be uninfected, decisions to avoid early mixed feeding and to shorten the duration of breastfeeding to 12 months can preserve many of the benefits of breastfeeding, while reducing the risk of HIV infection [11,43-45].

## The EID 'cascade' in resource-limited settings

Access to virological tests for EID has been limited in many resource-constrained settings, and pediatric providers have often relied on infant diagnosis algorithms that combine clinical symptoms with infant antibody testing [10,46-48]. As described above, these algorithms are neither sensitive nor specific in young infants [49], and HIV diagnosis and ART initiation often occur well after infancy [50,51]. Since 2005, PMTCT and pediatrics programs report rapidly expanding access to EID and infant ART [52]. Published and presented reports emphasize that DBS RNA [53,54] and DNA [1,3,28,31,55-71] PCR assays are feasible and acceptable for EID in resource-limited settings. However, these reports highlight that many infants are lost from care at each step in the EID cascade (Figure 1), including: infant presentation to care, test offer by healthcare professionals and test acceptance by parents/caregivers, specimen processing, result return to healthcare facilities and parents/caregivers, and linkage to care. Reported experiences of EID programs in resource-limited settings are summarized in Table 1. Many of these reports suggest high losses to follow-up at each step in the EID cascade, which likely have important impacts on the clinical outcomes, costs, and cost effectiveness of EID programs.

**Table 1:** Recent reports of early infant diagnostic testing programs in resource-limited settings: loss to follow-up at key steps in the 'cascade' of care

First author, year, reference	Location	Program, population	Findings
**Proportion of HIV-exposed infants undergoing EID testing:**			

Creek, 2008 [3]	Botswana	Pilot EID program: HIV-exposed infants attending well child visits, and inpatient and outpatient care settings	Of estimated 1,500 HIV-exposed children attending well child (clinic) visits, 1,297 (86%) were tested (total HIV-exposed no. not estimated for inpatient/outpatient settings)

Dow, IAS 2009 [71]	Malawi	Infants of known HIV-infected mothers offered EID at 6 weeks of age	Of 646 HIV-infected mothers, 338 (53%) presented for EID testing

Kimario, HAIM 2009 [62]	Tanzania	Ministry of Health-supported EID scale-up program: HIV-exposed infants age 1-9 months	Of 2,128 HIV-exposed infants identified, 2,089 (98%) were tested

Leroy, IAS 2009 [81]	Côte d'Ivoire	EID program: all infants attending immunization or outpatient clinics	Of 7,579 eligible, 3,013 (40%) offered EID testing, and 447 accepted (15% of offered, 6% of total)

Rollins, 2009 [68]	South Africa	All mothers/infants attending immunization clinics at 6, 10, or 14 weeks of age (median 7.7 weeks)	Of 646 mothers, 584 (90%) agreed to and underwent EID testing

Nuwagaba, 2010 [70]	Tanzania	Pilot EID program: HIV-exposed infants identified via maternal status or positive HIV antibody result	Of 510 HIV-exposed infants identified, 441 (87%) were tested

Braun, 2011 [110]	Malawi	Retrospective review of ANC, EID, and pediatric ART programs in Lilongwe, Malawi, 2004-2008	Of 14,669 HIV-exposed infants identified, 7,875 (54%) were tested

Hassan, 2011 [76]	Kenya	Retrospective review of HIV-exposed infants enrolled in an HIV clinic in Kilifi District, Kenya, 2006-2008	Of 233 HIV-exposed infants enrolled in care, 156 (67%) were tested

**Specimen collection and processing:**			
Creek, 2008 [3]	Botswana	As above	Of 1,931 samples, 27 (1.4%) unevaluable due to labeling errors

Khamadi, 2008 [61]	Kenya	Pilot EID program: HIV-exposed infants seen at 6 week immunization visit	Of 9,922 samples, 3 (0.03%) unevaluable due to errors in specimen collection or packaging

Kouakou, IAS 2008 [63]	Côte d'Ivoire	Pilot EID program in 25 PMTCT sites in 10 districts: HIV-exposed infants	Of 588 specimens, 92 (16%) unevaluable, and an additional 54 (9%) were not evaluated for unspecified reasons

Lofgren, 2009 [28]	Tanzania	DBS RNA PCR service for remote healthcare facilities	Of 223 samples, 27 (12%) 'lost with assay errors' and 7 (3%) specimens hemolyzed or of insufficient quantity

Menzies, 2009 [66]	Uganda	Pilot EID program: HIV-exposed infants age 6 weeks to 18 months	Of 820 samples, 32 (4%) unevaluable due to sample mislabeling, damage or loss or missing/inconsistent data entries

**Proportion of EID results returned**:			

Creek, 2008 [3]	Botswana	As above	Of 930 tests performed at well child (clinic) visits, 753 results (81%) returned to families. Of 38 infants with positive results at all sites, 34 results (90%) returned to families

Chouraya, IAS 2009 [59]	Swaziland	Pilot EID program: HIV-exposed infants, aged < 12 months, with positive PCR results	Of 124 infants with positive PCR results, 22 (18%) started ART before pilot program (result receipt inferred), and 50 (40%) were informed of results via pilot (total = 72 (58%) of 124). Additionally, 11 infants (9%) died before pilot and 41 (33%) were unable to be contacted; result receipt among these infants is unknown.

Kimario, HAIM 2009 [62]	Tanzania	As above	Of 2,089 infants tested, 1,331 results (64%) returned to facilities, and 774 results (37%) returned to families

Leroy, IAS 2009 [81]	Cote d'Ivoire	As above	Of 42 tested infants, 25 (60%) of families returned for results

Rollins, 2009 [68]	South Africa	As above	Of 584 infants tested, 332 mothers (57%) returned for results

Sundaram, IAS 2009 [69]	Swaziland	Retrospective review of HIV-infected infants diagnosed via DBS PCR at 15 clinical sites	Of 176 positive PCR results, 137 results (78%) returned to healthcare facility and 77 results (44%) returned to families

Nuwagaba, 2010 [70]	Tanzania	As above	Of 441 tested infants, 242 (55%) returned for results. Of 75 with positive PCR results, 51 (68%) returned for results; 7 (14%) of these children had died before result receipt. Of 361 with negative PCR results, 187 (52%) returned for results. Of five with indeterminate PCR results, four (80%) returned for results.

Hassan, 2011 [76]	Kenya	As above	Of 156 infants tested, 110 (71%) returned for results.

**Time to return of EID results to healthcare facilities or families/caregivers:**			

Creek, 2008 [3]	Botswana	As above	Specimen collection to results return at healthcare facilities: 9 days

Khamadi, 2008 [61]	Kenya	As above	Specimen collection to result return to families: range, 1-3 months. Individual components: Specimen collection to specimen receipt in laboratory: range, 6 hours to 3 weeks. Specimen receipt in laboratory to result dispatch from laboratory: 2006-2007: median 13.5 days, range 6-20 days; 2008: median 4 days.

Kouakou, IAS 2008 [63]	Côte d'Ivoire	As above	Specimen receipt in laboratory to result return to facilities: 4-8 weeks

Mahdi, IAS 2008 [65]	Swaziland	EID program and Public Health Unit: HIV-exposed infants aged < 18 months; n = 322	'Average turnaround time': 4-6 weeks

Kimario, HAIM 2009 [62]	Tanzania	As above	'Turnaround time': 1-3 months

Lofgren, 2009 [28]	Tanzania	As above	Specimen collection to result return to facilities: median 23 days, range 4-69 days (excluding single vacation period responsible for greatest delays: median 17 days, range 4-39 days). Individual components: Specimen collection to shipment from facilities: range 0-7 days. Transit, facilities to laboratories: median 1.5 days, range 1-2 days. Laboratory processing: median 13 days, range 1-51 days (excluding single vacation period as above: median 7 days, range 1-21 days). Completion of laboratory processing to shipment of results: range, 0-7 days. Transit, laboratories to facilities: median 1.5 days, range 1-2 days.

Rollins, 2009 [68]	South Africa	As above	Of 332 families returning for results, 160 (48%) returned at scheduled visit 2 weeks after testing. Of 172 returning at another time, 138 (80%) returned approximately 4 weeks after testing.

Nuwagaba, 2010 [70]	Tanzania	As above	Positive PCR results: median 5 weeks, range < 1-14 weeks. Negative PCR results: median 10 weeks, range < 1-21 weeks.

Hassan, 2011 [76]	Kenya	As above	'Waiting time to DBS results': median 1.7 months

**Proportion of identified HIV-infected infants linking to HIV care**:			

Creek, 2008 [3]	Botswana	As above	Of 34 PCR-positive infants receiving results, 22 (65%) were seen in ART clinic

Kimario, HAIM 2009 [62]	Tanzania	As above	Of 190 PCR-positive infants receiving results, 175 (92%) were referred to HIV care and treatment clinic, and 149 (78%) enrolled at clinic

Mahdi, IAS 2008 [65]	Swaziland	As above	Of 19 infants with positive PCR results, 13 (68%) linked to HIV care

Sundaram, IAS 2009 [69]	Swaziland	As above	Of 77 PCR-positive infants receiving results, 58 (75%) enrolled at ART clinic

Nuwagaba, 2010 [70]	Tanzania	As above	Of 52 PCR-positive infants receiving results, 42 (81%) were referred to HIV care (7 of 52 (14%) had died when result was received, and 3 of 52 (6%) died between result receipt and referral)

Braun, 2011 [110]	Malawi	As above	Of 1,084 infants with positive PCR results, 320 (30%) were traced to enrolment in an HIV care and treatment clinic

**Proportion of identified HIV-infected infants initiating cotrimoxazole:**			

Augustinova, IAS 2009 [89]	Cambodia	Retrospective review of EID among HIV-exposed infants aged < 18 months at PMTCT and pediatric inpatient and outpatient sites	Of 37 infants with positive PCR results, 13 (35%) initiated OI prophylaxis; an additional 14 (38%) initiated ART (if OI prophylaxis inferred, total = 27 of 37 (73%))

**Proportion of identified HIV-infected infants initiating ART:**			

Creek, 2008 [3]	Botswana	As above	Of 22 infants enrolled in HIV clinic, 17 (77%) initiated ART. These 17 infants represent 45% of the 38 infants with positive PCR results, and 50% of the 34 infants who received positive PCR results.

Mahdi, IAS 2008 [65]	Swaziland	As above	Of 13 PCR-positive infants linking to care, 5 (38%) initiated ART. These 5 infants represent 26% of the 19 infants with positive PCR results.

Augustinova, IAS 2009 [89]	Cambodia	As above	Of 37 infants with positive PCR results, 14 (38%) initiated ART

Chouraya, IAS 2009 [59]	Swaziland	As above	Of 124 infants with positive PCR results, 22 (18%) initiated ART before pilot. Through EID pilot program, 25 additional infants (20%) received results and initiated ART; total = 47 of 124 (38%). Additionally, 11 infants (9%) died before the pilot program and 41 (33%) were unable to be contacted through pilot; although unlikely, ART receipt among these infants is unknown.

Kimario, HAIM 2009 [62]	Tanzania	As above	Of 149 PCR-positive infants enrolled in care and treatment clinic, 68 (46%) initiated ART. These 68 infants represent 36% of the 190 results returned to families, and 22% of the 310 results returned to facilities.

Sundaram, IAS 2009 [69]	Swaziland	As above	Of 58 infants enrolled at ART clinic, 34 (59%) initiated ART. These 34 infants represent 19% of the 176 infants with positive PCR results.

Braun, 2011 [110]	Malawi	As above	Of 202 PCR-positive infants enrolled in a care and treatment clinic, 110 (55%) initiated ART at a median 2.5 months after enrollment

ANC = antenatal clinic; ART = antiretroviral therapy; CTX = cotrimoxazole; DBS = dried blood spot; EID = early infant diagnosis; HAIM = President's Emergency Plan for AIDS Relief (PEPFAR)-sponsored HIV/AIDS Implementers Meeting; IAS = International AIDS Society Conference; OI = opportunistic infection.

## Proportion of HIV-exposed infants undergoing EID testing

A large proportion of HIV-exposed infants never enter the EID pathway. To undergo EID testing, an infant must be brought by a caregiver to a healthcare facility, and healthcare providers must offer EID testing to the infant/caregiver pair. Most EID programs target infants for whom maternal HIV infection is already known (Table 1). Focusing testing efforts only on these known HIV-exposed infants may result in missed testing opportunities for more than half of all truly HIV-exposed infants [4,71-73].

Even when HIV-infected mothers are aware of their own HIV infection, this information may not be shared with pediatric providers, for example if the importance of disclosure is not emphasized, if mothers are reluctant to disclose their HIV infections, or if infants are brought to care by non-maternal caregivers [68,71,73]. Strategies to improve linkage between PMTCT and pediatric records are under investigation, and include child health cards indicating maternal HIV status [74] and integrated maternal and child health programs [75,76].

Such efforts may miss the large number of infants whose mothers are unaware of their own HIV infections. This may occur for many HIV-infected women, as a result of lack of HIV testing in pregnancy or false-negative HIV antibody tests during acute maternal infection [1]. Importantly, children of women with undiagnosed HIV infection are at substantial risk for HIV infection due to missed opportunities for PMTCT interventions [73,77], and those unknowingly infected in the peripartum or postpartum periods likely have the highest risk of MTCT [77,78]. When HIV diagnosis is unknown, healthcare providers may not consider EID testing necessary, or may feel uncomfortable offering an infant test that will reveal maternal HIV status [68].

To improve testing rates among both known and unknown HIV-exposed infants, several EID programs have broadened their target population to include sick infants presenting to care in inpatient or outpatient settings [3,55,79-81]. However, such programs offer testing when infants develop symptoms suggestive of HIV/AIDS, obviating the benefits of ART initiation before symptoms develop [33]. An innovative strategy of 'universal testing' of healthy infants at vaccination clinics (regardless of information about maternal HIV status) was found to be feasible and acceptable in South Africa, a high-HIV prevalence setting [68,82]. Importantly, parental acceptance of routine infant HIV testing at well child and vaccination visits may be substantially lower in lower-prevalence settings [81].

## Specimen collection and processing

There are also major challenges in EID specimen collection and processing (Table 1). Reported rates of unevaluable specimens vary widely, from 0.03% to 16% [61,64]. Reasons for unevaluable specimens include errors in specimen collection, labeling, packaging and data entry, as well as damage or loss of specimens and assay failures in the laboratory [3,28,61,63,66].

## Return of results to clinics and patients/caregivers

After EID assays are performed in the laboratory, results must next be returned to healthcare providers and then to families/caregivers. Rates of result return (37% to 90%) and time to result return (9 days to 21 weeks) range widely [3,62,70]. The time required for processing in the laboratory can be variable (1-51 days in a report from Tanzania) [28]. However, equally important events leading to lack of result return (Table 1) and delays in result return (Table 1) occur during the transmission of results from laboratory to healthcare providers and from providers to patients and families.

Currently, many central laboratories send paper-based EID results via commercial couriers, which may result in slow delivery [61,83]. Telephone-based and facsimile-based result return are limited by resource and internet availability, as well as laws regarding electronic transmission of health information [83,84]. Furthermore, health centers may lack systematic processes for following up test results. Because EID programs lie at the interface of PMTCT and pediatric programs [70] (in some cases, with separate health ministries overseeing each), there may be no clearly identified single agency responsible for ensuring that caregivers receive infant test results. Anecdotes describe single healthcare workers assuming this role; when these workers are absent, results may go unretrieved and unreturned [85,86]. In addition, since laboratory reports may be lost or delayed, EID results may not be available when caregivers return to receive them. The variability in result availability at patient return visits may be a more important reason for lack of patient result receipt than is failure of patients to keep follow-up appointments [76,83].

There is a clear need for improved medical informatics for laboratories and health centers [84]. Simple, inexpensive, and portable point-of-care DNA PCR and p24 antigen assays are also in development, but may be years from widespread use in remote settings [23,27,87]. In the interim, novel testing algorithms seek to minimize the need for return visits to receive results. For example, results of an antibody-based rapid HIV test (RHT) can be returned at an initial testing visit [66,68,70,79]. From 9-12 months of age, a negative RHT result can be reassuring with regard to both maternal and infant status, while a reactive result may motivate caregivers to return for the results of a PCR assay, which can be then performed on the same DBS sample used for the rapid test [55].

## Linkage to HIV care and treatment

Finally, delays and losses to follow-up between receipt of positive HIV test results and the initiation of HIV care and ART are additional missed opportunities to improve pediatric health. Pre-ART losses to program have been less well described in children than in adults [88]. Where available, program reports have demonstrated that up to 35% of infants diagnosed through EID programs may fail to link to HIV treatment clinics (Table 1) [3]. A large proportion of children enrolling in HIV care may also experience delays in ART initiation (in some cases leading to pre-ART mortality) [59], or lack of appropriate ART initiation [3,62,65,69,89]. These studies must be interpreted in view of changing ART initiation guidelines during the study periods; because ART was only recently recommended for all HIV-infected infants, programs operating before 2008 would not have sought to place 100% of HIV-infected infants on ART [6,10,32].

## Overall participation

An 'index of participation' can be calculated to reflect successful completion of the EID cascade (Table 2). The index of participation is derived by multiplying the proportions of infants with successful uptake at each step in the cascade, prior to and including any given step of interest. For example, using the highest reported values of uptake at each step from Table 1, an estimated 87.9% of HIV-exposed infants would be expected to undergoing EID testing and receive test results. Using the lowest published values, this proportion would be 1.9%. When extended to include linkage to HIV care and initiation of ART, the estimated rates of successful completion of the entire cascade range from 0.5% to 52.8%, highlighting that even under the best reported conditions, nearly half of HIV-infected infants may not link successfully to care and ART.

**Table 2:** Index of participation through each step in the early infant diagnosis (EID) cascade

Step in cascade	**Highest value from Table **1	**Lowest value from Table **1	**Cumulative index of participation through step in cascade (range, lowest to highest values from Table **1)
Proportion of exposed infants undergoing EID testing	98% [62]	6% [81]	

Specimen collection and processing	99.7% evaluable [61]	84% evaluable [64]	5.9% to 97.7%

Proportion of EID results returned	90% [3]	37% [62]	1.9% to 87.9%

Proportion of infected infants linking to care (of infected infants receiving results)	78% [62]	65% [3]	1.2% to 68.6%

Proportion of infected infants initiating ART (of infected infants receiving results and linking to care)	77% [3]	38% [65]	0.5% to 52.8%

For each step in the EID cascade, the highest and lowest reported values from Table 1 are highlighted. The index of participation is calculated as the product of successful uptake through each step in the cascade. For example, the highest value of uptake through 'specimen collection and processing' is calculated as the product of the highest value for the prior step ('proportion of exposed infants undergoing EID testing', 98%) and the highest value for the current step (99.7%); 0.98 × 0.997 = 0.9771. Results highlight that even with the highest reported uptake values, the total proportion of infected infants successfully accessing all steps in the care cascade is low, at 52.81%. As noted in the text, prior to 2008, fewer than 100% of HIV-infected infants would have been recommended to initiate ART, and the estimate of 1.4% to 68.6% of infected infants linking to care may be a better reflection of cascade completion. These calculations are conducted for purposes of discussion only, and require the assumption that the prevalence of HIV-infected infants among those not accessing each step in the cascade is equal to the prevalence among infants accessing care.

ART = antiretroviral therapy.

## Economic analyses of EID testing algorithms

Cost is often described as the principal barrier to the expansion of early infant diagnosis and ART [11], and a growing body of literature examines the current costs of EID programs. Here, we review published economic analyses of EID programs, focusing on three primary approaches: (1) costs to healthcare providers and HIV care programs, (2) costs to patients, and (3) cost effectiveness. The first approach, program-perspective or provider-perspective cost estimates, are valuable in planning short-term budgets for EID programs. However, these estimates omit important direct or indirect costs incurred by the families and caregivers of patients, which are included in the second approach. The third approach is the formal evaluation of cost effectiveness, which estimates the health and economic benefits obtained in return for investment in EID programs.

## Provider-perspective or program-perspective costs

When EID costs are reported, most authors describe the cost, per patient tested, to healthcare providers or health programs [3,11,23,61]. Although purchase prices of DNA PCR test kits may be variably negotiated between governments and producers, published per-patient test kit prices range from $8.00 in Botswana [3] to $50.00 in South Africa (years of currency not reported) [90]. In addition to test kits, a Kenyan pilot program highlights other important costs (2007 US$): insurance, freight, and taxes ($4 per specimen); filter papers ($2 per specimen); other reagents ($4 per specimen); courier services ($0.50 per specimen); and personnel time ($1 per specimen); combined with test kits costing $10.67 per patient, these costs led to a total per-test cost of $22.17 [61]. In Uganda, EID costs including personnel, supplies, and equipment totaled $23.90 to $24.01 (2007 US$), with test kits comprising 55% of total costs [66]; in Botswana, incorporation of the costs of test kits, laboratory products, technician time, and machine maintenance led to an estimated total cost of $19.60 per patient tested [3].

While these reports from Botswana, Kenya, and Uganda explicitly include many key costs [3,61,66], five key components of EID programs are difficult to cost accurately and are omitted from many other EID program reports:

### Transport costs

The costs of specimen transport from testing sites to processing laboratories, as well as the transport of results (electronically or on paper) back to providers, are infrequently described [28,61].

### Laboratory costs

Current assays for EID require substantial laboratory infrastructure, including not only PCR machines, laboratory space, and stable electricity, but also detailed and accurate systems for recording and delivering results. Reported assay costs usually include costs of disposable elements and reagents [61,66], but less often incorporate amortized costs of PCR machines or ongoing machine maintenance costs [3,23].

### Training costs

Training costs are rarely reported, including training of phlebotomists, laboratory technicians and counselors, for whom previous training may not have addressed ways to discuss results that reveal not only infant, but also maternal, HIV status [63,64,68].

### Quality control/quality assurance costs

Available cost estimates exclude the cost of ongoing quality control and quality assurance programs. Such programs are likely to be both time and labor intensive when conducted thoroughly, for example via shipment and processing of standardized 'control' specimens [91], but are clearly needed to prevent rejection of up to 16% of specimens due to poor specimen quality (Table 1) [64].

### Healthcare delivery systems

The economic impacts of variation in healthcare delivery systems are also rarely described, including anticipated reductions in per-test costs as greater numbers of infants are tested, reflecting economies of scale, and variations in cost between primary, district, and tertiary health centers. In addition, EID programs may be integrated into other maternal, neonatal, and child health programs. For example, return visits to receive EID test results are often scheduled alongside routine immunization visits [4]. An important consideration in EID cost analyses is the degree to which many of the costs described above are already included in existing maternal-child health program budgets.

## Patient or societal perspective costs

The second approach to estimating EID costs incorporates not only costs to healthcare providers and programs, but also costs to patients, family members, and society. This 'societal perspective' includes both direct costs (money spent by health programs or patients) and indirect costs, such as wages lost when patients miss work to attend clinic appointments or when illness limits economic productivity. While societal-perspective cost estimates are more difficult to calculate and may be less directly useful to program directors planning short-term budgets than program-perspective or provider-perspective estimates, they permit a more comprehensive evaluation of the economic impact of EID programs.

Sherman and colleagues in Johannesburg reported a cost analysis of an 'early diagnosis protocol', consisting of DBS PCR at 6 weeks of age, with a planned return visit to receive results at 3 months of age [4]. The early diagnosis protocol was compared to the standard diagnosis protocol at that time: HIV ELISA testing at 12 and 15-18 months. The early diagnosis protocol was estimated to identify 39% of all HIV-infected infants in the PMTCT program, compared to 15% identified by the standard protocol. In a provider-perspective cost analysis, the standard protocol cost $62.38 per patient (2003 US$) and the early diagnosis protocol cost $63.81 per patient. In a societal-perspective analysis, direct patient expenditures (travel fare, refreshments) and indirect costs, such as forfeited wages, were included. In this analysis, due to a large number of clinic visits averted, the early diagnosis protocol was cost saving compared to the standard protocol ($80.69 vs. $107.51 per patient). Extrapolating to all HIV-exposed infants in South Africa, the authors note that implementing the early diagnosis protocol would cost the health system over $400,000 per year, but would lead to societal savings of $7.5 million for those infants [4].

## Cost-effectiveness analysis

Because the costs associated with EID in Africa are high relative to antibody-based strategies for HIV diagnosis ($1 to $2 per test [11]), program planners and policy makers must evaluate whether the benefits of EID programs are 'worth' their greater costs. Cost-effectiveness analysis is a formal methodology used to address this question, incorporating not only costs, but also clinical outcomes, of alternative health interventions. By convention, both current and future costs and savings are included. Similarly, both short-term and long-term clinical benefits are quantified, most often in either years of life saved (YLS) or quality-adjusted years of life saved (QALY, which value each year lived in imperfect health less than each year in perfect health) [92].

Using the cost and effectiveness outcomes for two alternative healthcare strategies, one can calculate an incremental cost-effectiveness ratio (ICER). The difference in costs between the competing strategies is the numerator, and the difference in effectiveness, or the incremental health benefit, comprises the denominator. ICERs are conventionally reported in $/YLS or $/QALY. Because these units are not specific to any single health condition, they can help inform decisions among a variety of health interventions for a given population. In addition, ICERs in $/QALY can be compared to international standards of cost effectiveness, such as the WHO-supported Commission on Macroeconomics and Health recommendation that an intervention with an ICER less than the *per capita *GDP of a country be considered 'very cost effective' in that setting [93]. It is important to note that cost-effectiveness results are not intended to be the sole factor in health-related decision-making; issues of fairness, feasibility, and affordability may be of equal or greater importance.

The calculation of health benefits in YLS requires detailed information regarding both short-term and long-term survival associated with key possible health outcomes from the program under evaluation. For example, in an EID program, one would need estimates of the life expectancy of: (1) an HIV-infected child diagnosed and linked to HIV care through an EID program, (2) an HIV-infected child diagnosed and linked to care through a comparator program (for example, antibody testing at 15-18 months of age), (3) an HIV-exposed uninfected child, and (4) an HIV-unexposed child. Such life expectancy estimates, which may require long-term clinical studies or detailed computer simulation models to determine, are rarely available for resource-limited settings [94,95]. As a result, many cost-effectiveness analyses related to pediatric HIV prevention [96-99] or diagnosis [66,100,101] have used a cost-effectiveness outcome of 'cost/case of pediatric HIV prevented' or 'cost/case of pediatric HIV diagnosed'. Although there are no international cost-effectiveness standards denominated in these units, if several cost-effectiveness analyses use the same outcome measure, the 'cost/case diagnosed' of a newly examined testing strategy can be compared to the 'cost/case diagnosed' of strategies previously described or currently in use.

Only one published study has examined the cost effectiveness of EID programs in resource-limited settings: a population of known HIV-exposed children in Uganda [66]. Using both a computer model and program data, the authors examined DBS-based strategies of DNA PCR for all infants ('PCR') and RHT followed by DNA PCR if RHT results were reactive ('RHT→PCR'). In all scenarios, RHT→PCR was less expensive; it was also less effective in most. Among known HIV-exposed infants in a 'poor compliance' scenario (with a 43% index of participation that is similar to many programs, Table 1[4,59,68,71]), the ICER of the PCR algorithm compared to RHT→PCR was $539 per 'infant correctly diagnosed and informed of result'. If RHT→PCR were the current practice, adopting the PCR algorithm would be economically efficient if policymakers were willing to pay $539 to correctly diagnose one infant and inform him/her of test results. Because ICERs in such units are not directly comparable to other published cost-effectiveness outcomes, it remains challenging to answer the question, is the PCR algorithm cost effective in Uganda compared to the RHT→PCR algorithm? However, this detailed analysis demonstrates that the RHT→PCR algorithm may lead to nearly equivalent clinical outcomes and lower costs, and may become economically preferred in specific settings, for example where retention in care is high, breastfeeding is common, or HIV prevalence is low.

## Impact of the EID cascade on costs and cost effectiveness

Current EID cost and cost-effectiveness analyses, combined with recent programmatic reports (Table 1), highlight the key factors that may effect both feasibility and cost effectiveness of EID programs in resource-limited settings. These factors reflect key steps along the 'cascade' of events required for effective EID and delineate high-priority areas of future research that will be needed to comprehensively assess the cost effectiveness of EID programs.

### Proportion of HIV-exposed infants undergoing EID testing

The numbers of infants who present for initial testing will dramatically impact the total costs and total benefits of an EID program. However, an increase in the number of infants tested may not impact the cost effectiveness of an EID program compared to deferred clinical or antibody-based diagnosis, since greater costs and greater benefits will accrue in both programs, and the incremental differences may be small. An important exception may occur if the prevalence of HIV exposure or HIV infection differs among the additional infants presenting for EID, as compared to deferred diagnosis. To best assess the cost effectiveness of sick-infant or 'universal testing' strategies, a formal evaluation of the demographics and HIV prevalence even of those not undergoing HIV testing (perhaps via anonymous seroprevalence studies) would be useful.

### Improvements in result receipt and linkage to HIV care

The proportion of children and families who receive their EID test results and link to pediatric HIV care and ART will likely have a greater impact on the cost effectiveness than will the proportion of truly HIV-exposed infants who seek EID testing. This occurs because children who undergo an EID test and are then lost to follow-up incur the cost of testing without receiving any benefit from early HIV care. As described previously, several innovative approaches to improve result return have been investigated, including screening via rapid HIV tests, followed by confirmatory PCR assays.

### HIV prevalence and populations targeted by EID programs

The cost effectiveness of any screening program depends on the prevalence of disease in the screened population [102]. Menzies and colleagues found that the RHT→PCR algorithm, while cost saving, was slightly less effective than a strategy of PCR testing, in a population comprised entirely of HIV-exposed infants [66]. However, known HIV-exposed infants may not be the population in which an RHT→PCR algorithm would be most effective or cost effective. Among very young HIV-exposed infants, negative RHTs are by definition false negatives with regard to maternal status and are therefore more likely to be false negatives with regard to infant infection status. False negativity may result from inherent test insensitivity, processing errors, or the 'window period' between postnatal infection and infant antibody production [6,10]. Among known HIV-exposed infants, then, routine PCR testing is more effective, and may also be cost effective. Rollins and colleagues investigated the RHT→PCR algorithm in a population in which it may be more cost effective: 'universal testing' at vaccination clinics [68,82].

As proof of concept [66,82] and feasibility and acceptability studies [68], both Menzies *et al*. and Rollins *et al*. processed DBS specimens for RHT [66] or ELISA [68,82] and PCR off site, and were therefore unable to return antibody results to caregivers at the same visit on which samples were obtained. Additional evaluations of the RHT/PCR algorithm are needed in populations of infants without known HIV exposure, with specific focus on the proportions of infants/caregivers receiving immediate RHT results, receiving delayed PCR results, and linking to HIV care.

### Long-term clinical and economic impacts of early infant diagnosis and care

To comprehensively assess the cost effectiveness of EID compared to traditional deferred diagnostic strategies, it will be critical to understand the clinical events and costs associated with early and late diagnosis and therapy. These factors may have greater impact on EID cost effectiveness than the comprehensive costs of laboratory assays including transport, equipment, training, and quality assurance. The immediate costs for PCR-based EID are higher than for deferred antibody-based testing [11]. Medication costs associated with early diagnosis will increase further if infants link to HIV services and early ART. Considering short-term benefits, such as the number of infants diagnosed, does not capture the larger clinical benefits of early diagnosis and therapy [33,103]. In addition, EID and early ART initiation will likely avert a large number of opportunistic infections and deaths [33,103], for which care is expensive; in the short term, this may offset a proportion of the upfront costs of EID programs [104]. For example, substantial cost savings were observed over a 10-month period in South Africa when ART was initiated immediately after EID, compared to ART deferred until the development of signs or symptoms of AIDS [34]. Data to inform clinical outcomes for HIV-exposed and HIV-infected children in various resource-limited settings are needed. As such data become available, health policy models [66,95] can integrate data from multiple sources, highlight future research priorities by identifying the most influential data parameters, and assist in informing HIV testing and treatment guidelines.

## Conclusions

Access to early infant diagnosis of HIV infection is improving in resource-limited settings, but key barriers exist to testing, result receipt, and linkage to care. Infants of mothers who are unaware of their own HIV infections or who did not access PMTCT services may be the most difficult population to capture, but may also be the population with the greatest need for EID. The benefits of successful EID are substantial, not only for HIV-infected infants, in whom morbidity and mortality can be reduced with early ART initiation, but also for HIV-negative infants, their caregivers, and PMTCT and pediatric providers. Costs are a frequently cited barrier to EID expansion. However, more detailed cost-effectiveness analyses will be important to assess the degree to which the benefits of EID programs render them 'worth' their costs, and long-term data and model-based projections can further inform future clinical and economic benefits. Innovative approaches to improve rates of testing, result receipt, and linkage to care will have lasting impacts on the health of HIV-exposed infants and the value of EID programs in resource-limited settings.

## Competing interests

The authors declare that they have no competing interests.

## Authors' contributions

ALC conceived the study, conducted literature review, data extraction, and interpretation of results, and drafted the manuscript. JP conducted literature review and data extraction. JP, LRA, KAF, RPW, and VL participated in the design of the study and interpretation of study results. All authors critically reviewed the manuscript and contributed to manuscript drafting, and all authors read and approved the final submitted manuscript.

## Pre-publication history

The pre-publication history for this paper can be accessed here:

http://www.biomedcentral.com/1741-7015/9/59/prepub
